# Is the Game Over or Starting Again? The Role of the Transplant Team in Genetic Counseling for Adult Sickle Cell Disease Recipients

**DOI:** 10.4274/tjh.2016.0355

**Published:** 2017-06-01

**Authors:** Pelin Aytan, Çiğdem Gereklioğlu, Mahmut Yeral, Aslı Korur, Süheyl Asma, İlknur Kozanoğlu, Hakan Özdoğu, Can Boğa

**Affiliations:** 1 Başkent University Training and Research Hospital, Bone Marrow and Stem Cell Transplantation Center, Clinic of Hematology, Adana, Turkey; 2 Başkent University Faculty of Medicine, Department of Family Medicine, Adana, Turkey

**Keywords:** Adult, sickle cell anemia, Genetic counseling, Transplantation

## To The Editor,

Hematopoietic stem cell transplantation (HSCT) with non-myeloablative regimens has become more feasible and it is currently performed more frequently in adult patients with sickle cell disease (SCD) [1]. Hsieh [1] reported successful outcomes for adults with a regimen of alemtuzumab and low-dose total body irradiation (TBI) in transplants from human leukocyte antigen (HLA)-matched sibling donors. We have obtained sustained full chimerism in 13 adults with protocols including antithymocyte globulin (ATG) and busulfan without any significant complications (unpublished data). For unrelated donors, the ongoing STRIDE study uses conditioning regimens containing ATG, fludarabine, and busulfan. Bolaños-Meade et al. [2] achieved a cure with HLA-haploidentical transplants performed with ATG, fludarabine, and low-dose TBI. Given that these protocols may allow assisted or spontaneous pregnancies, fertility issues need to be examined [3] because the patient may think that he/she is completely cured after allogeneic transplantation and that he/she no longer carries the hemoglobin S (*Hb S*) gene. This erroneous belief may lead to the delivery of a homozygous *Hb SS* infant if the partner has a high risk of being a *Hb S* gene carrier when marriages of close relatives occur, as in some populations like Eti-Turks.

SCD is the most common hereditary disease worldwide. The impaired microcirculation caused by rigid erythrocytes leads to considerable mortality and severe morbidity if not managed appropriately [4]. The only proven curative therapy is HSCT. However, this treatment carries high risk for infertility as a major complication for young patients [5]. Factors predisposing to infertility in these patients include delayed puberty, priapism, and gonadal dysfunction with increased abnormal spermatozoa, seminal vesicle and prostate gland abnormalities, decreased ejaculate volume, lower testosterone levels, and low sperm counts in males [6,7,8]. Conditioning regimens including alemtuzumab and low-dose TBI are reported not to irreparably impair spermatogenesis, although there are insufficient data about non-myeloablative conditioning including ATG and busulfan [9].

When young SCD patients want to marry after full recovery with transplantation, some of them tend to hide their disease intentionally or due to a lack of awareness. A complete blood count and hemoglobin electrophoresis test as part of the screening done before marriage may give normal results. Consequently, affected germ cells may be overlooked. This may result in giving birth to affected children. The transplant team is responsible for providing sufficient information about these issues.
